# Bis(μ-5-carb­oxy­benzene-1,3-dicarboxyl­ato)-κ^3^ *O* ^1^,*O* ^1′^:*O* ^3^;κ^3^ *O* ^3^:*O* ^1^,*O* ^1′^-bis­[(2-phenyl-1,3,7,8-tetra­aza­cyclo­penta­[*l*]phenanthrene-κ^2^ *N* ^7^,*N* ^8^)lead(II)]

**DOI:** 10.1107/S1600536810045812

**Published:** 2010-11-24

**Authors:** Jing Chen, Xiang-Cheng Wang, Chun-Xiang Li

**Affiliations:** a School of Chemistry and Chemical Engineering, Jiangsu University, Zhenjiang 212013, People’s Republic of China

## Abstract

In the title compound, [Pb_2_(C_9_H_4_O_6_)_2_(C_19_H_12_N_4_)_2_], the Pb^II^ atom is five-coordinated by two N atoms from a chelating 2-phenyl-1*H*-1,3,7,8-tetra­aza­cyclo­penta­[*l*]phenanthrene (*L*) ligand and three O atoms from two Hbtc ligands (H_3_btc is benzene-1,3,5-tricarb­oxy­lic acid), resulting in a distorted PbN_2_O_3_ coordination. Two Pb^II^ atoms are bridged by the Hbtc ligands, forming a discrete centrosymmetric dinuclear complex. Inter­molecular N—H⋯O and O—H⋯O hydrogen bonds and π–π inter­actions between the pyridine and imidazole rings, and between the pyridyl rings of the *L* ligands [centroid–centroid distances = 3.600 (6) and 3.732 (6) Å] lead to a three-dimensional supra­molecular structure.

## Related literature

For general background to the structures and potential applications of supra­molecular architectures, see: Che *et al.* (2008 ▶). For a related structure, see: Liu *et al.* (2009 ▶). For the ligand synthesis, see: Steck & Day (1943 ▶).
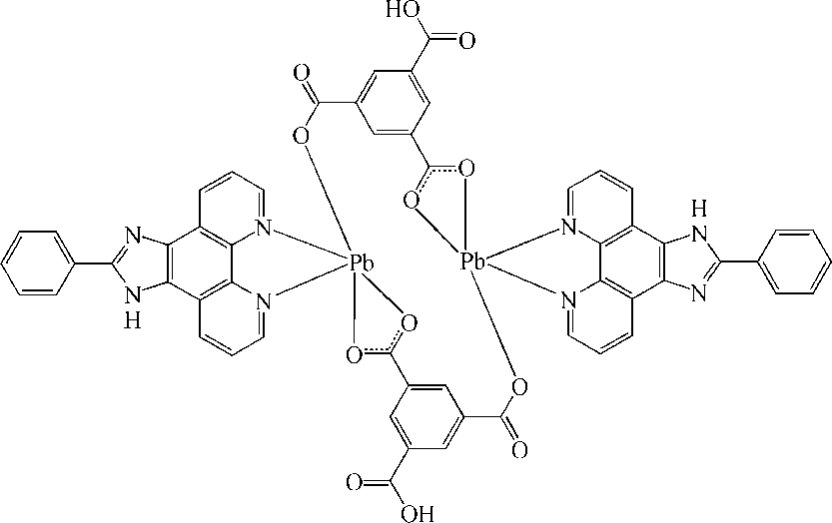


## Experimental

### 

#### Crystal data

[Pb_2_(C_9_H_4_O_6_)_2_(C_19_H_12_N_4_)_2_]
*M* *_r_* = 1423.28
Triclinic, 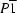

*a* = 9.2776 (3) Å
*b* = 11.4409 (5) Å
*c* = 12.2764 (6) Å
α = 73.820 (4)°
β = 72.754 (4)°
γ = 68.680 (4)°
*V* = 1137.74 (9) Å^3^
*Z* = 1
Mo *K*α radiation
μ = 7.47 mm^−1^
*T* = 292 K
0.30 × 0.26 × 0.23 mm

#### Data collection

Oxford Diffraction Gemini R Ultra CCD diffractometer
Absorption correction: multi-scan (*CrysAlis RED*; Oxford Diffraction, 2006 ▶) *T*_min_ = 0.122, *T*_max_ = 0.179
5399 measured reflections
3979 independent reflections
3547 reflections with *I* > 2σ(*I*)
*R*_int_ = 0.021

#### Refinement

*R*[*F* ^2^ > 2σ(*F* ^2^)] = 0.024
*wR*(*F* ^2^) = 0.055
*S* = 1.00
3979 reflections
352 parameters
H-atom parameters constrained
Δρ_max_ = 0.82 e Å^−3^
Δρ_min_ = −1.28 e Å^−3^

### 

Data collection: *CrysAlis CCD* (Oxford Diffraction, 2006 ▶); cell refinement: *CrysAlis RED* (Oxford Diffraction, 2006 ▶); data reduction: *CrysAlis RED*; program(s) used to solve structure: *SHELXTL* (Sheldrick, 2008 ▶); program(s) used to refine structure: *SHELXTL*; molecular graphics: *SHELXTL* and *DIAMOND* (Brandenburg, 1999 ▶); software used to prepare material for publication: *SHELXTL*.

## Supplementary Material

Crystal structure: contains datablocks global, I. DOI: 10.1107/S1600536810045812/hy2360sup1.cif

Structure factors: contains datablocks I. DOI: 10.1107/S1600536810045812/hy2360Isup2.hkl

Additional supplementary materials:  crystallographic information; 3D view; checkCIF report

## Figures and Tables

**Table 1:** Selected bond lengths (Å)

Pb—N1	2.561 (4)
Pb—N2	2.449 (3)
Pb—O1^i^	2.342 (3)
Pb—O3	2.530 (3)
Pb—O6^i^	2.903 (3)

Symmetry code: (i) 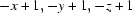.

**Table 2:** Hydrogen-bond geometry (Å, °)

*D*—H⋯*A*	*D*—H	H⋯*A*	*D*⋯*A*	*D*—H⋯*A*
N4—H4⋯O2^ii^	0.86	1.97	2.815 (5)	169
O4—H4*A*⋯O6^iii^	0.82	1.83	2.653 (4)	177

Symmetry codes: (ii) 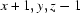; (iii) 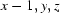.

**Fig. 1.**
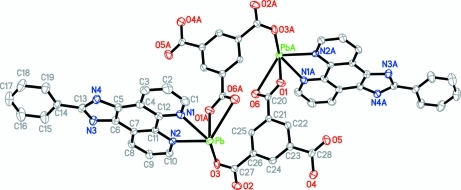
Molecular structure of the title compound. Displacement ellipsoids are drawn at the 50% probability level. H atoms have been omitted for clarity. [Symmetry code: (A) 1-x, 1-y, 1-z.]

**Fig. 2.**
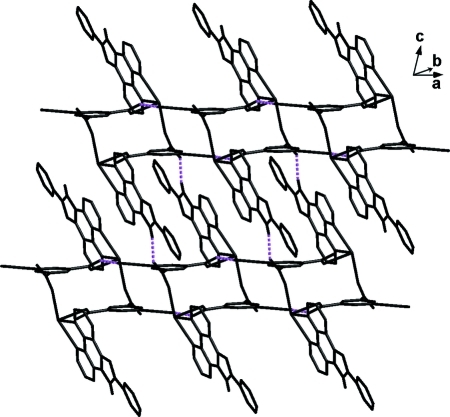
Crystal packing of the title compound, with hydrogen bonds indicated by dotted lines.

## supplementary crystallographic information

### Comment

The design and construction of supramolecular architectures have received considerable attention in recent years, mostly motivated by their intriguing structural features and potential applications in catalysis, magnetism, molecular adsorption, non-linear optics and molecular sensing (Che *et al.*, 2008). 2-Phenyl-1*H*-1,3,7,8-tetraazacyclopenta[*l*]phenanthrene (*L*) as an important phenanthroline derivative possesses fruitful aromatic systems and is a good candidate for the construction of metal–organic supramolecular architectures (Liu *et al.*, 2009). In this paper, we selected benzene-1,3,5-tricarboxylic acid (H_3_btc) as a linker and *L* as a secondary ligand, resulting in the title complex.

In the title compound, the Pb^II^ atom is surrounded by two N atoms from one *L* ligand and three O atoms from two Hbtc ligands (Fig. 1). The neighboring two Pb^II^ atoms are bridged by the two Hbtc ligands, forming a sixteen-membered ring with a long Pb···Pb distance of 8.3037 (5) Å. Adjacent dimers are further linked through intermolecular N—H···O and O—H···O hydrogen bonds and π–π interactions between the pyridyl and imidazole rings and between the pyridyl rings of the *L* ligands [centroid–centroid distances = 3.600 (6) and 3.732 (6) Å], leading to a three-dimensional supramolecular structure (Fig. 2).

### Experimental

The *L* ligand was synthesized according to the literature method (Steck & Day, 1943). The title compound was synthesized under hydrothermal conditions. A mixture of *L* (0.060 g, 0.2 mmol), H_3_btc (0.042 g, 0.2 mmol), Pb(NO_3_)_2_ (0.066 g, 0.2 mmol) and water (10 ml) in a mole ratio of 1:1:1:5000 was placed in a 25 ml Teflon-lined autoclave and heated for 3 d at 433 K under autogenous pressure. Upon cooling and opening the bomb, yellow block-shaped crystals were obtained, washed with H_2_O and dried in air (yield: 65% based on Pb).

### Refinement

H atoms on C and N atoms were positioned geometrically and refined as riding atoms, with C—H = 0.93 and N—H = 0.86 Å and *U*_iso_(H) = 1.2*U*_eq_(C,N). H atom of the carboxyl group was located from a difference Fourier map and refined as riding, with O—H = 0.82 Å and *U*_iso_(H) = 1.5*U*_eq_(O).

### Crystal data

**Table:**

[Pb_2_(C_9_H_4_O_6_)_2_(C_19_H_12_N_4_)_2_]	*Z* = 1
*M**_r_* = 1423.28	*F*(000) = 684
Triclinic, *P*1	*D*_x_ = 2.077 Mg m^−^^3^
Hall symbol: -P 1	Mo *K*α radiation, λ = 0.71073 Å
*a* = 9.2776 (3) Å	Cell parameters from 6223 reflections
*b* = 11.4409 (5) Å	θ = 4.3–29.1°
*c* = 12.2764 (6) Å	µ = 7.47 mm^−^^1^
α = 73.820 (4)°	*T* = 292 K
β = 72.754 (4)°	Block, yellow
γ = 68.680 (4)°	0.30 × 0.26 × 0.23 mm
*V* = 1137.74 (9) Å^3^	

### Data collection

**Table:**

Oxford Diffraction Gemini R Ultra CCD diffractometer	3979 independent reflections
Radiation source: fine-focus sealed tube	3547 reflections with *I* > 2σ(*I*)
graphite	*R*_int_ = 0.021
ω scans	θ_max_ = 25.0°, θ_min_ = 4.3°
Absorption correction: multi-scan (*CrysAlis RED*; Oxford Diffraction, 2006)	*h* = −11→11
*T*_min_ = 0.122, *T*_max_ = 0.179	*k* = −12→13
5399 measured reflections	*l* = −14→14

### Refinement

**Table:**

Refinement on *F*^2^	Primary atom site location: structure-invariant direct methods
Least-squares matrix: full	Secondary atom site location: difference Fourier map
*R*[*F*^2^ > 2σ(*F*^2^)] = 0.024	Hydrogen site location: inferred from neighbouring sites
*wR*(*F*^2^) = 0.055	H-atom parameters constrained
*S* = 1.00	*w* = 1/[σ^2^(*F*_o_^2^) + (0.0349*P*)^2^] where *P* = (*F*_o_^2^ + 2*F*_c_^2^)/3
3979 reflections	(Δ/σ)_max_ = 0.006
352 parameters	Δρ_max_ = 0.82 e Å^−^^3^
0 restraints	Δρ_min_ = −1.28 e Å^−^^3^

### Fractional atomic coordinates and isotropic or equivalent isotropic displacement parameters (Å^2^)

**Table:**

	*x*	*y*	*z*	*U*_iso_*/*U*_eq_	
C1	0.7189 (5)	0.2900 (4)	0.0863 (4)	0.0311 (11)	
H1	0.6208	0.3528	0.0892	0.037*	
C2	0.8282 (6)	0.2868 (5)	−0.0199 (4)	0.0340 (11)	
H2	0.8024	0.3455	−0.0862	0.041*	
C3	0.9727 (5)	0.1970 (4)	−0.0252 (4)	0.0284 (10)	
H3	1.0473	0.1940	−0.0952	0.034*	
C4	1.0091 (5)	0.1086 (4)	0.0756 (4)	0.0230 (9)	
C5	1.1546 (5)	0.0094 (4)	0.0827 (4)	0.0235 (9)	
C6	1.1849 (5)	−0.0717 (4)	0.1856 (4)	0.0219 (9)	
C7	1.0700 (4)	−0.0637 (4)	0.2914 (3)	0.0183 (8)	
C8	1.0944 (5)	−0.1447 (4)	0.3980 (4)	0.0236 (9)	
H8	1.1921	−0.2058	0.4036	0.028*	
C9	0.9727 (5)	−0.1323 (4)	0.4929 (4)	0.0257 (10)	
H9	0.9871	−0.1848	0.5643	0.031*	
C10	0.8272 (5)	−0.0415 (4)	0.4835 (4)	0.0239 (9)	
H10	0.7448	−0.0361	0.5491	0.029*	
C11	0.9200 (5)	0.0296 (4)	0.2884 (3)	0.0188 (8)	
C12	0.8917 (5)	0.1180 (4)	0.1795 (3)	0.0207 (9)	
C13	1.3926 (5)	−0.1295 (4)	0.0540 (4)	0.0251 (9)	
C14	1.5482 (5)	−0.2019 (4)	−0.0049 (4)	0.0248 (9)	
C19	1.6161 (5)	−0.1588 (5)	−0.1198 (4)	0.0335 (11)	
H19	1.5631	−0.0815	−0.1614	0.040*	
C18	1.7622 (6)	−0.2314 (5)	−0.1714 (4)	0.0407 (13)	
H18	1.8074	−0.2031	−0.2483	0.049*	
C17	1.8423 (6)	−0.3458 (6)	−0.1105 (5)	0.0493 (15)	
H17	1.9410	−0.3941	−0.1462	0.059*	
C26	0.2977 (4)	0.2718 (4)	0.6601 (3)	0.0183 (8)	
C24	0.1360 (5)	0.3197 (4)	0.6642 (3)	0.0219 (9)	
H24	0.0784	0.2638	0.6764	0.026*	
C23	0.0600 (5)	0.4498 (4)	0.6502 (4)	0.0206 (9)	
C22	0.1453 (5)	0.5317 (4)	0.6363 (4)	0.0223 (9)	
H22	0.0948	0.6194	0.6255	0.027*	
C21	0.3059 (4)	0.4849 (4)	0.6381 (4)	0.0203 (9)	
C25	0.3821 (4)	0.3546 (4)	0.6483 (3)	0.0191 (9)	
H25	0.4901	0.3226	0.6472	0.023*	
C27	0.3789 (5)	0.1300 (4)	0.6694 (4)	0.0204 (9)	
C28	−0.1124 (5)	0.5072 (4)	0.6481 (4)	0.0230 (9)	
C20	0.3893 (5)	0.5775 (4)	0.6332 (4)	0.0224 (9)	
C16	1.7755 (6)	−0.3883 (6)	0.0038 (5)	0.0504 (15)	
H16	1.8298	−0.4651	0.0452	0.060*	
C15	1.6285 (6)	−0.3171 (5)	0.0564 (4)	0.0380 (12)	
H15	1.5834	−0.3462	0.1330	0.046*	
N1	0.7482 (4)	0.2074 (3)	0.1833 (3)	0.0232 (8)	
N2	0.8010 (4)	0.0378 (3)	0.3847 (3)	0.0191 (7)	
N4	1.2883 (4)	−0.0273 (3)	−0.0012 (3)	0.0244 (8)	
H4	1.3037	0.0068	−0.0739	0.029*	
N3	1.3343 (4)	−0.1584 (4)	0.1674 (3)	0.0259 (8)	
O3	0.4905 (4)	0.0925 (3)	0.5863 (3)	0.0346 (8)	
O2	0.3312 (4)	0.0561 (3)	0.7562 (3)	0.0312 (7)	
O1	0.3317 (3)	0.6937 (3)	0.5876 (3)	0.0281 (7)	
O6	0.5074 (3)	0.5389 (3)	0.6780 (3)	0.0380 (8)	
O5	−0.1757 (3)	0.6197 (3)	0.6241 (3)	0.0371 (8)	
O4	−0.1880 (3)	0.4204 (3)	0.6780 (3)	0.0395 (8)	
H4A	−0.2815	0.4561	0.6755	0.059*	
Pb	0.537207 (17)	0.188377 (15)	0.371460 (14)	0.02330 (7)	

### Atomic displacement parameters (Å^2^)

**Table:**

	*U*^11^	*U*^22^	*U*^33^	*U*^12^	*U*^13^	*U*^23^
C1	0.032 (2)	0.023 (2)	0.035 (3)	−0.0044 (19)	−0.012 (2)	0.000 (2)
C2	0.042 (3)	0.029 (3)	0.024 (2)	−0.007 (2)	−0.014 (2)	0.007 (2)
C3	0.036 (2)	0.028 (2)	0.017 (2)	−0.011 (2)	−0.0024 (19)	0.000 (2)
C4	0.026 (2)	0.023 (2)	0.020 (2)	−0.0090 (18)	−0.0063 (18)	−0.0022 (18)
C5	0.024 (2)	0.024 (2)	0.023 (2)	−0.0074 (18)	−0.0021 (18)	−0.0083 (19)
C6	0.022 (2)	0.024 (2)	0.021 (2)	−0.0061 (18)	−0.0059 (18)	−0.0061 (19)
C7	0.020 (2)	0.021 (2)	0.016 (2)	−0.0078 (16)	−0.0030 (17)	−0.0051 (17)
C8	0.026 (2)	0.018 (2)	0.022 (2)	−0.0015 (17)	−0.0045 (18)	−0.0030 (18)
C9	0.031 (2)	0.024 (2)	0.015 (2)	−0.0024 (19)	−0.0059 (19)	−0.0004 (18)
C10	0.027 (2)	0.024 (2)	0.016 (2)	−0.0069 (18)	−0.0001 (18)	−0.0027 (19)
C11	0.022 (2)	0.020 (2)	0.017 (2)	−0.0097 (17)	−0.0022 (17)	−0.0051 (17)
C12	0.028 (2)	0.019 (2)	0.016 (2)	−0.0071 (17)	−0.0076 (18)	−0.0024 (17)
C13	0.022 (2)	0.029 (2)	0.022 (2)	−0.0086 (18)	−0.0009 (18)	−0.007 (2)
C14	0.023 (2)	0.032 (3)	0.021 (2)	−0.0108 (19)	0.0004 (18)	−0.010 (2)
C19	0.030 (2)	0.039 (3)	0.027 (2)	−0.006 (2)	−0.002 (2)	−0.009 (2)
C18	0.031 (3)	0.059 (4)	0.026 (3)	−0.010 (2)	0.003 (2)	−0.013 (3)
C17	0.033 (3)	0.056 (4)	0.050 (4)	0.003 (3)	−0.003 (3)	−0.027 (3)
C26	0.020 (2)	0.020 (2)	0.013 (2)	−0.0057 (16)	−0.0014 (16)	−0.0024 (17)
C24	0.023 (2)	0.023 (2)	0.022 (2)	−0.0109 (18)	−0.0027 (18)	−0.0046 (18)
C23	0.019 (2)	0.022 (2)	0.022 (2)	−0.0048 (17)	−0.0064 (17)	−0.0042 (18)
C22	0.022 (2)	0.017 (2)	0.024 (2)	−0.0020 (17)	−0.0048 (18)	−0.0033 (18)
C21	0.018 (2)	0.018 (2)	0.023 (2)	−0.0045 (16)	−0.0051 (17)	−0.0022 (18)
C25	0.0150 (19)	0.018 (2)	0.020 (2)	−0.0027 (16)	−0.0007 (16)	−0.0034 (17)
C27	0.020 (2)	0.019 (2)	0.025 (2)	−0.0054 (17)	−0.0094 (19)	−0.0044 (19)
C28	0.017 (2)	0.026 (2)	0.024 (2)	−0.0057 (18)	−0.0034 (18)	−0.0049 (19)
C20	0.017 (2)	0.024 (2)	0.025 (2)	−0.0067 (17)	0.0019 (18)	−0.0089 (19)
C16	0.044 (3)	0.046 (3)	0.047 (3)	0.006 (3)	−0.010 (3)	−0.014 (3)
C15	0.039 (3)	0.036 (3)	0.027 (3)	0.003 (2)	−0.007 (2)	−0.008 (2)
N1	0.0248 (18)	0.0207 (19)	0.0231 (19)	−0.0057 (15)	−0.0088 (15)	−0.0008 (16)
N2	0.0187 (16)	0.0216 (18)	0.0160 (18)	−0.0080 (14)	−0.0015 (14)	−0.0024 (15)
N4	0.0246 (18)	0.027 (2)	0.0182 (19)	−0.0072 (15)	0.0006 (15)	−0.0051 (16)
N3	0.0224 (18)	0.030 (2)	0.021 (2)	−0.0047 (16)	−0.0026 (15)	−0.0052 (17)
O3	0.0308 (17)	0.0241 (17)	0.0301 (18)	0.0034 (14)	0.0087 (14)	−0.0088 (15)
O2	0.0413 (18)	0.0236 (17)	0.0254 (17)	−0.0120 (14)	−0.0078 (14)	0.0033 (14)
O1	0.0315 (16)	0.0210 (16)	0.0350 (18)	−0.0103 (13)	−0.0128 (14)	−0.0013 (14)
O6	0.0233 (16)	0.0293 (18)	0.067 (2)	−0.0096 (14)	−0.0204 (17)	−0.0045 (17)
O5	0.0235 (16)	0.0238 (18)	0.060 (2)	−0.0008 (13)	−0.0140 (16)	−0.0062 (16)
O4	0.0156 (14)	0.0305 (18)	0.071 (2)	−0.0068 (13)	−0.0128 (16)	−0.0040 (17)
Pb	0.01918 (9)	0.02098 (10)	0.02834 (11)	−0.00717 (6)	−0.00471 (7)	−0.00173 (7)

### Geometric parameters (Å, °)

**Table:**

C1—N1	1.328 (6)	C18—H18	0.9300
C1—C2	1.394 (7)	C17—C16	1.383 (8)
C1—H1	0.9300	C17—H17	0.9300
C2—C3	1.357 (6)	C26—C25	1.388 (6)
C2—H2	0.9300	C26—C24	1.388 (5)
C3—C4	1.403 (6)	C26—C27	1.508 (5)
C3—H3	0.9300	C24—C23	1.382 (6)
C4—C12	1.412 (6)	C24—H24	0.9300
C4—C5	1.421 (6)	C23—C22	1.379 (6)
C5—N4	1.371 (5)	C23—C28	1.497 (5)
C5—C6	1.380 (6)	C22—C21	1.393 (5)
C6—N3	1.377 (5)	C22—H22	0.9300
C6—C7	1.416 (6)	C21—C25	1.386 (5)
C7—C8	1.404 (6)	C21—C20	1.502 (6)
C7—C11	1.416 (5)	C25—H25	0.9300
C8—C9	1.359 (6)	C27—O2	1.239 (5)
C8—H8	0.9300	C27—O3	1.262 (5)
C9—C10	1.386 (6)	C28—O5	1.196 (5)
C9—H9	0.9300	C28—O4	1.327 (5)
C10—N2	1.324 (5)	C20—O6	1.253 (5)
C10—H10	0.9300	C20—O1	1.269 (5)
C11—N2	1.356 (5)	C16—C15	1.380 (7)
C11—C12	1.463 (6)	C16—H16	0.9300
C12—N1	1.350 (5)	C15—H15	0.9300
C13—N3	1.330 (6)	Pb—N1	2.561 (4)
C13—N4	1.374 (5)	Pb—N2	2.449 (3)
C13—C14	1.462 (6)	Pb—O1^i^	2.342 (3)
C14—C15	1.387 (6)	Pb—O3	2.530 (3)
C14—C19	1.394 (6)	Pb—O6^i^	2.903 (3)
C19—C18	1.376 (6)	O4—H4A	0.8200
C19—H19	0.9300	N4—H4	0.8600
C18—C17	1.379 (8)		
			
N1—C1—C2	123.1 (4)	C23—C24—C26	120.5 (4)
N1—C1—H1	118.5	C23—C24—H24	119.8
C2—C1—H1	118.5	C26—C24—H24	119.8
C3—C2—C1	119.1 (4)	C22—C23—C24	119.3 (4)
C3—C2—H2	120.4	C22—C23—C28	117.9 (4)
C1—C2—H2	120.4	C24—C23—C28	122.8 (4)
C2—C3—C4	119.6 (4)	C23—C22—C21	121.0 (4)
C2—C3—H3	120.2	C23—C22—H22	119.5
C4—C3—H3	120.2	C21—C22—H22	119.5
C3—C4—C12	117.7 (4)	C25—C21—C22	119.2 (4)
C3—C4—C5	125.7 (4)	C25—C21—C20	122.2 (3)
C12—C4—C5	116.5 (4)	C22—C21—C20	118.6 (4)
N4—C5—C6	106.2 (3)	C21—C25—C26	120.1 (3)
N4—C5—C4	131.0 (4)	C21—C25—H25	119.9
C6—C5—C4	122.8 (4)	C26—C25—H25	119.9
N3—C6—C5	110.6 (4)	O2—C27—O3	123.3 (4)
N3—C6—C7	127.5 (4)	O2—C27—C26	118.9 (4)
C5—C6—C7	121.8 (4)	O3—C27—C26	117.7 (4)
C8—C7—C11	118.2 (4)	O5—C28—O4	123.7 (4)
C8—C7—C6	124.0 (4)	O5—C28—C23	123.3 (4)
C11—C7—C6	117.8 (3)	O4—C28—C23	113.0 (4)
C9—C8—C7	118.9 (4)	O6—C20—O1	122.8 (4)
C9—C8—H8	120.6	O6—C20—C21	119.3 (4)
C7—C8—H8	120.6	O1—C20—C21	117.8 (3)
C8—C9—C10	120.1 (4)	C17—C16—C15	120.1 (5)
C8—C9—H9	119.9	C17—C16—H16	120.0
C10—C9—H9	119.9	C15—C16—H16	120.0
N2—C10—C9	122.6 (4)	C16—C15—C14	120.0 (5)
N2—C10—H10	118.7	C16—C15—H15	120.0
C9—C10—H10	118.7	C14—C15—H15	120.0
N2—C11—C7	121.1 (3)	C1—N1—C12	118.4 (4)
N2—C11—C12	119.2 (3)	C1—N1—Pb	123.8 (3)
C7—C11—C12	119.7 (4)	C12—N1—Pb	117.3 (3)
N1—C12—C4	122.0 (4)	C10—N2—C11	119.1 (3)
N1—C12—C11	116.8 (4)	C10—N2—Pb	120.9 (3)
C4—C12—C11	121.2 (3)	C11—N2—Pb	119.9 (2)
N3—C13—N4	112.4 (4)	C5—N4—C13	106.3 (3)
N3—C13—C14	123.3 (4)	C5—N4—H4	126.9
N4—C13—C14	124.3 (4)	C13—N4—H4	126.9
C15—C14—C19	119.8 (4)	C13—N3—C6	104.5 (3)
C15—C14—C13	118.3 (4)	C27—O3—Pb	130.6 (3)
C19—C14—C13	121.9 (4)	C20—O1—Pb^i^	107.3 (2)
C18—C19—C14	119.5 (5)	C28—O4—H4A	109.5
C18—C19—H19	120.3	O1^i^—Pb—N2	75.71 (10)
C14—C19—H19	120.3	O1^i^—Pb—O3	86.66 (11)
C19—C18—C17	120.8 (5)	N2—Pb—O3	79.48 (10)
C19—C18—H18	119.6	O1^i^—Pb—N1	78.54 (11)
C17—C18—H18	119.6	N2—Pb—N1	66.07 (10)
C18—C17—C16	119.8 (5)	O3—Pb—N1	144.85 (10)
C18—C17—H17	120.1	O6^i^—Pb—N1	79.58 (10)
C16—C17—H17	120.1	O6^i^—Pb—N2	119.29 (10)
C25—C26—C24	119.8 (4)	O6^i^—Pb—O1^i^	48.50 (10)
C25—C26—C27	121.0 (3)	O6^i^—Pb—O3	113.57 (10)
C24—C26—C27	119.3 (4)		

Symmetry codes: (i) −*x*+1, −*y*+1, −*z*+1.

### Hydrogen-bond geometry (Å, °)

**Table:**

*D*—H···*A*	*D*—H	H···*A*	*D*···*A*	*D*—H···*A*
N4—H4···O2^ii^	0.86	1.97	2.815 (5)	169
O4—H4A···O6^iii^	0.82	1.83	2.653 (4)	177

Symmetry codes: (ii) *x*+1, *y*, *z*−1; (iii) *x*−1, *y*, *z*.
